# Genome-Wide Identification and Characterization of *Calcium-Dependent Protein Kinase* (*CDPK*) and *CDPK-Related Kinase* (*CRK*) Gene Families in *Medicago truncatula*

**DOI:** 10.3390/ijms22031044

**Published:** 2021-01-21

**Authors:** Pengcheng Zhao, Yajie Liu, Weiyi Kong, Jiayi Ji, Tianyu Cai, Zhenfei Guo

**Affiliations:** College of Grassland Science, Nanjing Agricultural University, Nanjing 210095, China; 2016220006@njau.edu.cn (P.Z.); 2018120001@njau.edu.cn (Y.L.); T2017076@njau.edu.cn (W.K.); 15517125@njau.edu.cn (J.J.); 15517132@njau.edu.cn (T.C.)

**Keywords:** genome-wide analysis, *Medicago truncatula*, CDPK, CRK, *cis*-acting elements, expression pattern, subcellular localization

## Abstract

Calcium-dependent protein kinase (CDPK or CPK) and CDPK-related kinase (CRK) play an important role in plant growth, development, and adaptation to environmental stresses. However, their gene families had been yet inadequately investigated in *Medicago truncatula*. In this study, six *MtCRK* genes were computationally identified, they were classified into five groups with *MtCDPKs* based on phylogenetic relationships. Six pairs of segmental duplications were observed in *MtCDPK* and *MtCRK* genes and the *Ka/Ks* ratio, an indicator of selection pressure, was below 0.310, indicating that these gene pairs underwent strong purifying selection. *Cis*-acting elements of morphogenesis, multiple hormone responses, and abiotic stresses were predicted in the promoter region. The spatial expression of *MtCDPKs* and *MtCRKs* displays diversity. The expression of *MtCDPKs* and *MtCRKs* could be regulated by various stresses. MtCDPK4, 14, 16, 22, and MtCRK6 harbor both N-myristoylation site and palmitoylation site and were anchored on plasma membrane, while MtCDPK7, 9, and 15 contain no or only one N-acylation site and were distributed in cytosol and nucleus, suggesting that the N-terminal acylation sites play a key role in subcellular localization of MtCDPKs and MtCRKs. In summary, comprehensive characterization of *MtCDPKs* and *MtCRKs* provide a subset of candidate genes for further functional analysis and genetic improvement against drought, cold, salt and biotic stress.

## 1. Introduction

Calcium is a ubiquitous second messenger in plant cells involving in growth, development, and responses to environmental stresses [1]. The external signals lead to a transient increased free Ca^2+^ concentration in the cytosol. Ca^2+^ is bound with calcium binding proteins (CBP), or Ca^2+^ sensors, which decode the signals and lead to cellular alterations in biochemical and physiological processes [2,3,4]. There are five types of Ca^2+^ sensors in plants, including calmodulin (CaM), calmodulin-like protein (CML), calcineurin B-like protein (CBL), calcium/calmodulin-dependent protein kinase (CCaMK) [5], and calcium-dependent protein kinase (CDPK) [6]. Compared to CaM, CML, and CBL, CDPKs directly transduce Ca^2+^ signal into phosphorylation cascades, which makes CDPKs have a dual function: Ca^2+^ sensors and responders [7].

CDPKs exist in higher plants, green algae, oomycetes, and protists, but absent in animals and fungi [8], while CRKs are only observed in plants [9]. CDPK harbors four domains: an N-terminal variable domain, a protein kinase domain, an autoinhibitory junction region, and a calmodulin-like domain (CaM-LD) which commonly containing four EF-hands [10]. CRK has a similar protein structure to CDPK with a degenerative CaM-LD, which causes the inability to bind Ca^2+^ [7]. There is generally an N-myristoylation site and a palmitoylation site in the N-terminal variable domain. N-myristoylation is irreversible and provides a loose membrane association, while palmitoylation is reversible and confers stable membrane anchoring [11]. The membrane binding ability of CDPKs is decreased when the palmitoylation or myristoylation site is mutated [12].

CDPKs and CRKs participate in regulation of multiple physiological processes. They are involved in pollen tube elongation [13], seedling and leaf development [14], shoot and root hairs development [15,16], root growth and gravitropism [17,18], flowering [19], seed development [20], and senescence-related cell death [21]. CDPKs and CRKs are involved in the responses to abiotic stresses. For example, CDPKs are involved in drought or salt stress adaptation through inducing ABF-mediated ABA-responsive gene expression and regulating ABA-induced anion channel (SLAC1, SLAH3) to modulate stomatal closure in *Arabidopsis* [22,23]. CDPKs activate the enzymatic reactive oxygen species (ROS) scavenging pathways to regulate drought and salt adaptation. Catalase CAT3 is phosphorylated by AtCPK8 for activating its activity in *Arabidopsis* [24], while ascorbate peroxidase *OsAPX2*/*OsAPX8* and NADPH oxidase *OsrbohI* expression is regulated by OsCPK12 in rice [25]. Moreover, vacuolar K^+^ channel TPK1 is phosphorylated by AtCPK3 to regulate the cytosolic K^+^/Na^+^ balance in responses to salt stress [26]. OsCPK17 participates in cold stress response through interacting with aquaporin OsPIP2;1/OsPIP2;6 and sucrose-phosphate synthase OsSPS4 [27]. GmCDPKSK5 in response to high temperature and humidity stress during soybean seed development through targeting translationally controlled tumor protein GmTCTP [28]. AtCRK1 is involved in maintaining cellular redox homeostasis and osmotic balance under continuous illumination and salt stress, respectively [18,29]. AtCRK1 is a positive regulator of thermotolerance by phosphorylation of AtHSFA1a to increase heat-shock transcription factor (HSF) DNA binding to the heat-shock element (HSE) [30]. CDPKs and CRKs have been reported in responses to biotic stresses. AtCPK1 affects the accumulation of salicylic acid (SA) and further induces expression of SA-regulated defense genes against fungus and bacterial pathogens [31]. AtCPK5, AtCPK6, and AtCPK11 affect *Botrytis cinereal* infection via inducing ethylene production through modulating the transcriptional level of ethylene biosynthesis enzyme ACC synthase *ACS2* and *ACS6* [32]. AtCPK5 phosphorylates NADPH oxidase homolog D (RBOHD) and leads to the production of extracellular H_2_O_2_, which is crucial for accelerating rapid signal propagation to defense response activation [33]. SlCRK6 is a positive regulator of *Pst* DC3000 and *Sclerotinia sclerotiorum* resistance in tomato [34]. MtCDPK8, 16, and 19 were reported to regulate root nodule development via symbiotic interaction in *M. truncatula* [35,36,37]. CDPK and CRK families have been demonstrated as key regulators in various signaling pathways in many plant species [38,39]. However, comprehensive bioinformatics analysis in *M. truncatula*, a legume model plant, had been yet inadequately investigated. Although 25 *MtCDPK* genes were identified and characterized [40], they were not named using a standard procedure and were not characterized for gene duplication, *cis*-acting elements, and subcellular localization. In addition, *MtCRK* genes have not been investigated.

The objectives of this study were to investigate the gene structure, conserved motifs, phylogenetic relationship, chromosome distribution, gene duplication, and *cis*-acting regulatory elements of *MtCDPK* and *MtCRK* gene families in *M. truncatula* and to analyze their spatial expression profiles and response to abiotic and biotic stresses. In addition, the subcellular localization of selected MtCDPKs and MtCRKs were analyzed to explore the role of N-terminal acylation in protein localization.

## 2. Results

### 2.1. Identification of MtCRK Genes

A genome-wide analysis of *MtCRK* gene families was performed based on *M. truncatula* genome sequences using HMMER3.0, SMART, and CDD software. A total of six *MtCRKs* were perceived from *M. truncatula* genome. They and previously identified *MtCDPKs* were denominated as *MtCDPK1* to *MtCDPK24* and *MtCRK1* to *MtCRK6*, respectively, depending on their chromosomal positions (Table 1). Except for the CaM-like domain consisting of zero to three EF-hands, MtCRKs share the similar domain with MtCDPKs (Table 1).

The gene length of *MtCRKs* varies from 4143 bp (*MtCRK6*) to 6710 bp (*MtCRK1*), encoding protein length varies from 581 (MtCRK6) to 596 (MtCRK5) amino acids (aa). The molecular weight (MW) varies from 65.15 (MtCRK6) to 66.80 kDa (MtCRK4), while the isoelectric point (pI) varies from 8.49 (MtCRK3) to 9.07 (MtCRK2), indicating that MtCRKs are alkaline. The grand average of hydropathy (GRAVY) ranges from −0.415 (MtCRK5) to −0.305 (MtCRK4), indicating that MtCRKs are hydrophilic. All MtCRKs contain both predicted N-terminal myristoylation and palmitoylation sites. In addition, most MtCDPKs harbor these predicted sites, while MtCDPK7 contains only a predicted palmitoylation site and MtCDPK15 contains a predicted myristoylation site. MtCDPK9, 10 and 23 were predicted to have neither myristoylation nor palmitoylation site (Table 1).

### 2.2. Phylogenetic and Chromosomal Distribution of MtCDPKs and MtCRKs

**Figure 1:**
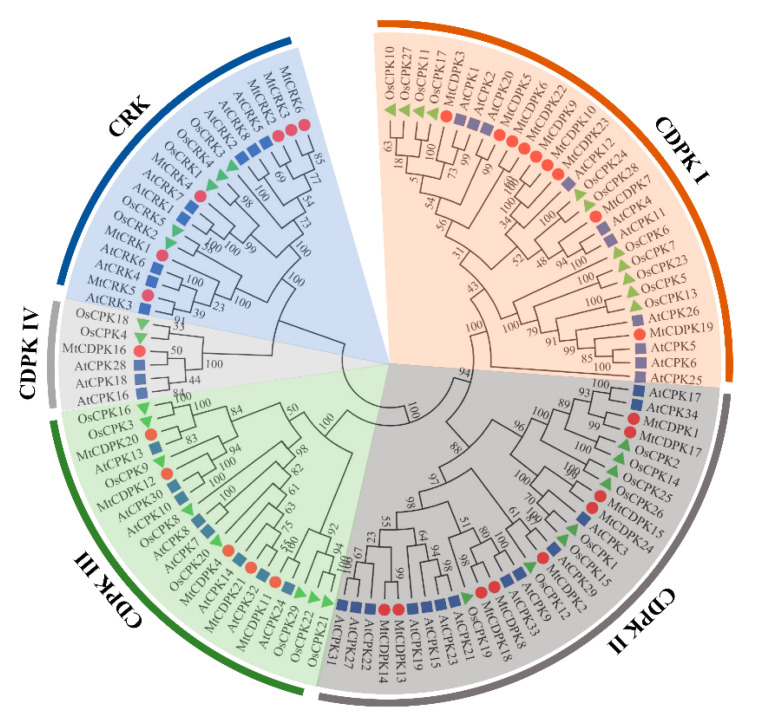
Phylogenetic relationships of CDPK and CRK proteins in *M. truncatula* (red circle), *A. thaliana* (blue square), and *O. sativa* (green triangle). The tree was constructed using MEGA7.0 software by the Neighbor-Joining method. The numbers next to the branch showed the 1000 bootstrap replicates expressed in percentage. The phylogenetic groups of MtCDPKs and MtCRKs were marked by different colors and legends.

A neighbor-joining (NJ) phylogenic tree was constructed using CDPK and CRK sequences from *M. truncatula*, *Arabidopsis*, and rice. The sequences of CDPK and CRK were listed in Table S1. All proteins were clustered into five groups, including four groups of CDPK (CDPK I to CDPK IV) and one group of CRK (Figure 1).

**Figure 2:**
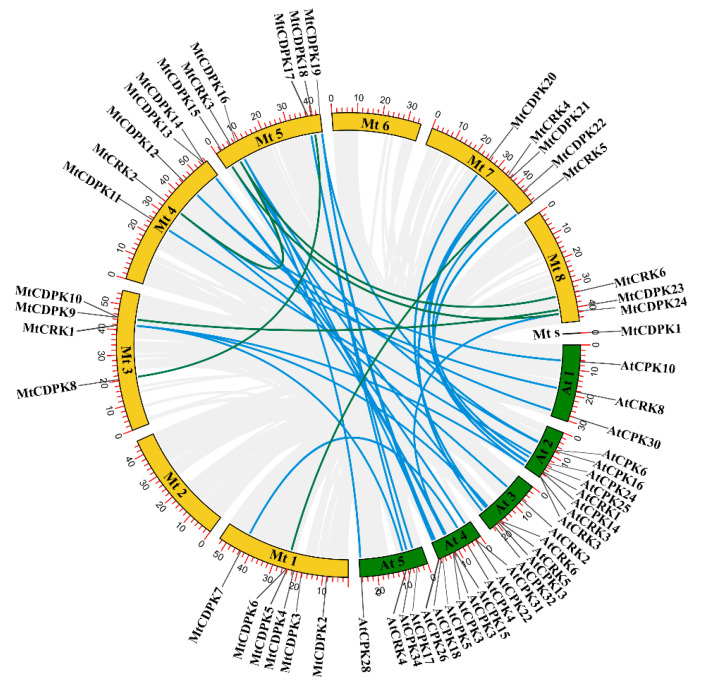
The collinear correlation of *MtCDPKs* and *MtCRKs* was displayed between *M. truncatula* and *Arabidopsis* genomes. The *M. truncatula* and *Arabidopsis* chromosomes were represented by yellow and green boxes, respectively. Collinear relationship of *CDPKs and CRKs* between *M. truncatula* and *Arabidopsis* indicated with blue lines, and within the *M. truncatula* genome indicated with green lines. s: *M. truncatula* scaffold0028.

The chromosomal location of *MtCDPKs* and *MtCRKs* was analyzed using TBtools software and gene annotation information. The results showed that *MtCDPKs* and *MtCRKs* are distributed on six chromosomes (Figure 2). Furthermore, the collinear correlation was calculated between *M. truncatula* and *A. thaliana* species to investigate the evolutionary history of *CDPKs* and *CRKs*. A total of 29 collinear gene pairs were found between *M. truncatula* and *A. thaliana* (Figure 2 and Table S2), indicating that these genes might have existed before the divergence between *M. truncatula* and *A. thaliana*. In addition, six gene pairs (*MtCDPK6*/*22*, *MtCDPK8*/*18*, *MtCDPK9*/*23*, *MtCDPK15*/*24, MtCRK2*/*3,* and *MtCRK3*/*6*) were found to be paralogous, indicating that they belonged to segmental duplications (Table 2). The selection pressure of the gene pairs was analyzed by computing the number of nonsynonymous substitutions per nonsynonymous site (*Ka*) and the number of synonymous substitutions per synonymous site (*Ks*) substitution rates (Table 2). The *Ka*/*Ks* value, an indicator for the selection history of these paralogous gene pairs, was below 0.310 (Table 2), indicating that these gene pairs underwent strong purifying selection in the evolutionary process, and led to the function of these gene pairs to be relatively conserved.

### 2.3. Analysis of Gene Structure and Conserved Motifs of MtCDPKs and MtCRKs

**Figure 3:**
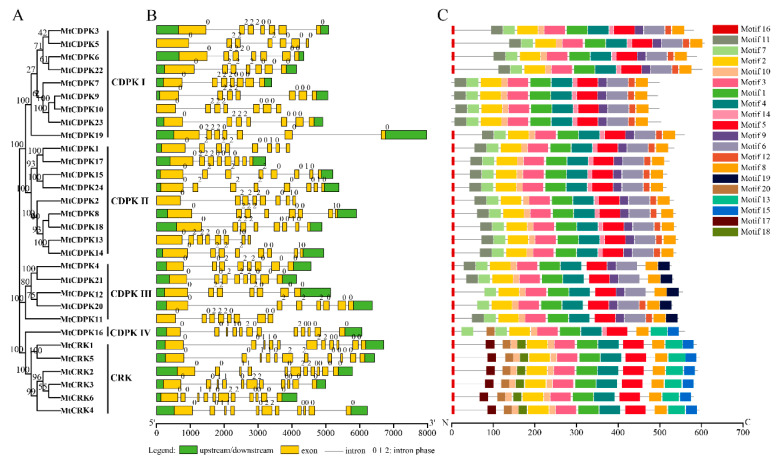
Phylogenetic relationship, gene structure, and conserved motifs of CDPKs and CRKs in *M. truncatula*. (**A**) The phylogenetic tree of MtCDPK and MtCRK proteins was constructed using MEGA7.0 software by the Neighbor-Joining method (1000 bootstrap replicates). (**B**) The gene structure of *MtCDPK* and *MtCRK* genes. The green boxes, yellow boxes, and black lines represent UTR, exon, and introns, respectively. Intron phases 0, 1, and 2 were labeled at the beginning of each intron. (**C**) The conserved motifs of MtCDPK and MtCRK proteins were identified using MEME. The motifs were indicated by different colored boxes and their numbers were listed on the right.

The phylogenetic tree of MtCDPKs and MtCRKs was constructed (Figure 3A), which was consistent with that in Figure 1. Gene structure was analyzed based on the sequence in untranslated region (UTR), exon, and intron using TBTools. All genes have a long initial exon, followed by multiple short exons varying from seven to twelve in numbers, *MtCRKs* harbor more exons than *MtCDPKs* except for *MtCDPK16* (Figure 3B). Interestingly, the exon-intron structure within the same group exhibits high similarity. For instance, all the genes in group CRK contain the identical number of exons and the same type of intron phase. *MtCRKs* had a similar intron-exon pattern with the members in CDPK IV group (Figure 3B).

The conserved motifs of MtCDPKs and MtCRKs were analyzed using the MEME program. Twenty conserved motifs varying from 8 to 50 aa. in lengths were observed. Only motif 16 was observed to be in the variable N-terminal domain, while the myristoylation and palmitoylation sites were observed in the motif 16 in all MtCDPKs and MtCRKs except for MtCDPK15. The kinase domain showed conserved, and many motifs upstream the motif 5 were observed in the kinase domain. An auto-inhibitory junction region was existed in the motif 5, and the EF-hands were observed in the motif 5, 6, 8, 9, and 13. The same conserved motifs were possessed by the members within the same group. The group-specific motifs were mainly observed at the N-terminal and C-terminal of proteins. For example, motifs 13, 15, 17, 18, and 20 were present specifically in the members of CRK group except for MtCDPK16, while the motif 19 was in the members of CDPK III group. The above motifs were also present in the same group of CRK or CDPK in *Arabidopsis* and rice (Figure 3C and Figure S1, and Table S3).

### 2.4. Cis-Acting Elements in the Promoter Region of MtCDPKs and MtCRKs

**Figure 4:**
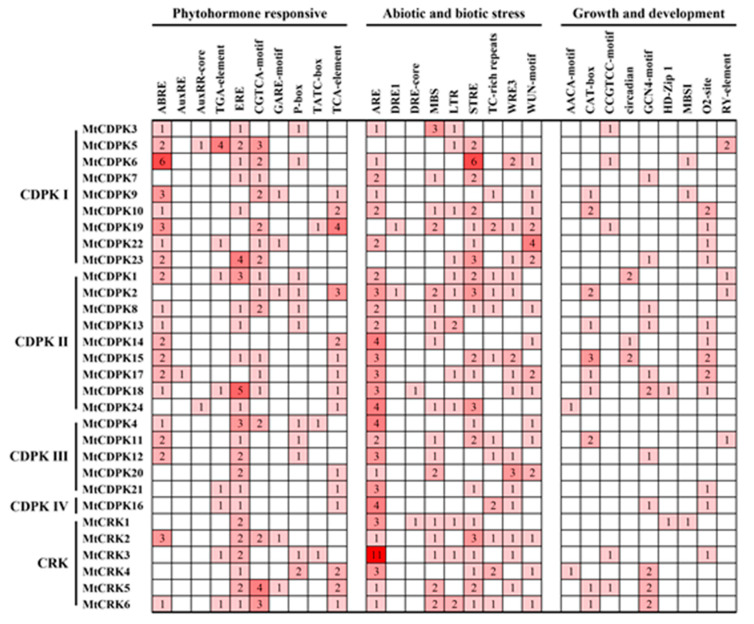
*Cis*-acting regulatory elements in the promoter region of *MtCDPK* and *MtCRK* genes. According to the function annotation, the elements were divided into the following three main categories: phytohormone responsive, abiotic and biotic stresses, and plant growth and development. The numbers and the depth of red represent the frequency of the elements that occur in the promoter region.

To gain insight into the potential function and regulatory mechanisms of the genes during plant development and responses to various stresses, the recognized putative *cis*-acting regulatory elements were analyzed from 2.0 kb DNA sequence in the promoter region of *MtCDPK* and *MtCRK* genes using the PlantCARE database. Various types of *cis*-acting regulatory elements were shown in Figure 4 and Table S4. There exist multiple phytohormone responsive elements including ABA response element (ABRE), auxin-responsive element (AuxRR, AuxRR-core, and TGA-element), ethylene-responsive element (ERE), MeJA-responsive element (CGTCA-motif), GA-responsive element (GARE-motif, P-box, and TATC-box), and salicylic acid-responsive element (TCA-element), suggesting that *MtCDPKs* and *MtCRKs* expression were regulated by different phytohormones. A variety of elements related to light-responsive elements were observed. The stress-responsive elements were found in the promoter of some *MtCDPKs* and *MtCRKs*, such as anaerobic induction element (ARE), dehydration-responsive element (DRE1 and DRE-core), drought-inducibility element (MBS), low-temperature-responsive element (LTR), defense and stress-responsive element (TC-rich repeats), stress-responsive element (STRE) and wound-responsive element (WRE3 and WUN-motif). Multiple growth and development elements were investigated, including endosperm-expression element (AACA-motif and GCN4-motif), meristem-expression element (CAT-box and CCGTCC-motif), circadian element, palisade mesophyll cells-expression element (HD-Zip 1), flavonoid biosynthetic genes regulation element (MBSI), zein metabolism regulation element (O2-site), and seed-specific regulation element (RY-element). Overall, two phytohormone responsive elements (ABRE and ERE) and two stress responsive elements (ARE and STRE) were detected in the promoter regions of most genes. But auxin-responsive elements, gibberellin-responsive elements, dehydration-responsive elements, and most of plant growth and development elements were found in the promoter regions of specific genes. The results indicated that *MtCDPKs* and *MtCRKs* are involved in plant growth and development as well as environmental stress responses.

### 2.5. Spatial Expression Profiles of MtCDPK and MtCRK Genes

**Figure 5:**
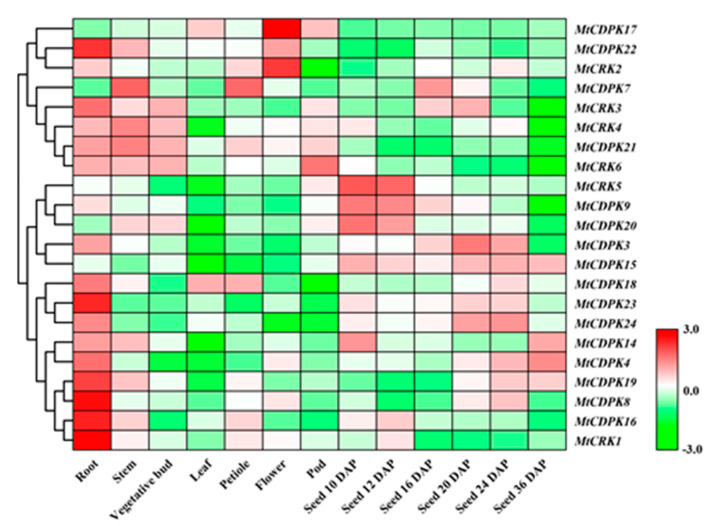
Expression patterns of *MtCDPK* and *MtCRK* genes in different developmental tissues. The microarray data were normalized based on the mean expression value of each gene in all organs analyzed. The heat map portrayed the relative expressions after log_2_ transformed. The corresponding group of each gene was shown on the left. DAP, days after pollination.

The spatial expression pattern of *MtCDPK* and *MtCRK* genes was analyzed based on microarray data from the *Medicago truncatula* Gene Expression Atlas (MtGEA, https://mtgea.noble.org/v3/). *MtCDPK7*, *17*, *22*, and *MtCRK3*, *6* transcripts level was lower than other genes. *MtCDPK8*, *16*, *18*, *19*, *22*, *23*, and *MtCRK1* transcript major observed in roots. *MtCDPK17* and *MtCRK2* were preferentially expressed in flower, while *MtCDPK14*, *MtCDPK20*, and *MtCRK4* transcript were low in leaf. *MtCRK5*, *MtCDPK9*, and *MtCDPK20* transcript were decreased during seed development, while *MtCDPK4* and *MtCDPK19* were increased (Figure 5, Table S5). The results suggested that *MtCDPK* and *MtCRK* genes might have diverse functions in different tissues. Unfortunately, no information on eight *MtCDPK* genes (*MtCDPK1*, *2*, *5*, *6*, *10*, *11*, *12*, and *13*) was available from MtGEA.

### 2.6. Expression Patterns of MtCDPK and MtCRK Genes in Response to Abiotic and Biotic Stresses

**Figure 6:**
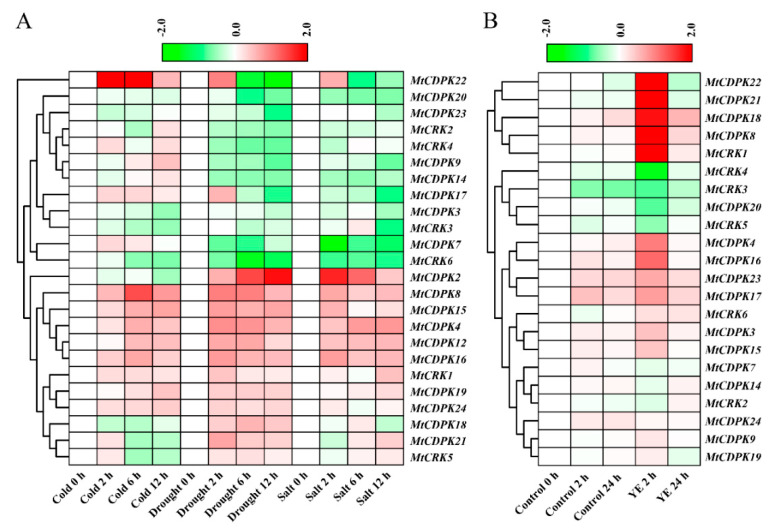
Expression patterns of *MtCDPK* and *MtCRK* genes under different abiotic treatments (**A**) and biotic treatment (**B**). The transcript levels after treatments were rescaled relative to that untreated time (at 0 h) when calculating the relative expression levels. The heat map portrayed the relative expressions after log_2_ transformed. The corresponding group of each gene was shown on the left. YE, yeast elicitor.

To analyze expression profiles of *MtCDPKs and MtCRKs* under abiotic stresses, available RNA-seq data were retrieved from NCBI (GEO database). Fragments per kilobase of exon per million fragments mapped (FPKM) values of *MtCDPK1*, *5*, *6*, *10*, *11*, and *13* were lower than 1, which were considered to be barely expressed and not employed for analysis. *MtCDPK4*, *8*, *15*, *16,* and *22* transcripts were quickly increased after 2 h of cold treatment. *MtCDPK2* transcript was increased continuously under drought stress, *MtCDPK4*, *8*, *12*, *15*, *16*, *21*, and *22* transcripts were increased and peaked after 2 h of drought treatment, whereas *MtCDPK22* transcript was decreased at 6 h. In contrast, *MtCDPK23* and *MtCRK6* were downregulated after drought treatment. In response to salt treatment, *MtCDPK2*, *4*, *8*, *12*, and *16* transcripts were upregulated after salt stress, while *MtCRK6*, *MtCDPK7*, and *MtCDPK17* transcripts were downregulated (Figure 6A and Table S6). The microarray data of treatment with yeast elicitor (YE) was used to analyze the responses of *MtCDPKs* and *MtCRKs* to the pathogen. *MtCDPK4*, *8*, *16*, *18*, *21*, *22*, and *MtCRK1* were upregulated significantly at 2 h after treatment with yeast elicitor, whereas *MtCRK4* was downregulated (Figure 6B and Table S7). The results indicated that *MtCDPK* and *MtCRK* genes showed diverse expression patterns under various stresses and may be involved in the regulation of abiotic and biotic stress responses. It is interesting that several *MtCDPKs* respond to multiple stresses. *MtCDPK4*, *8*, and *16* were upregulated by cold, drought, salt, and pathogen, and *MtCDPK12* were upregulated by cold, drought, and salt, while *MtCDPK15* was upregulated by cold and drought. They are probably involved in plant adaptation to multiple stresses.

### 2.7. Subcellular Localization of Selected MtCDPKs and MtCRKs

**Figure 7:**
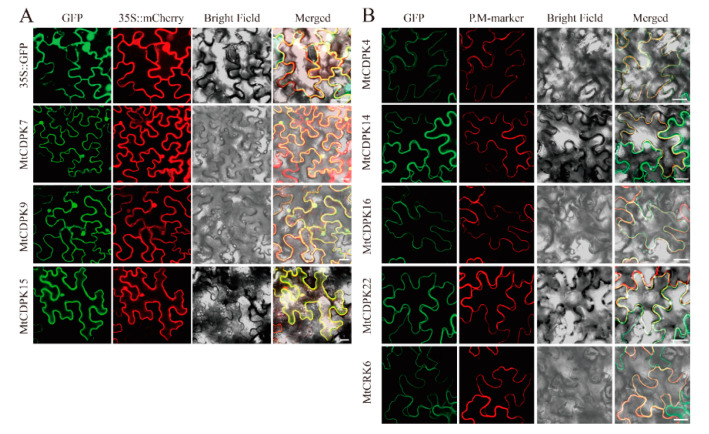
Subcellular localization of MtCDPKs- and MtCRKs-GFP fusion proteins in combination with 35S::mCherry (**A**) and PM-marker (**B**) by using the epidermal cells of *N. benthamiana*. The tobacco injected with P.M-marker was observed after plasmolysis. PM-marker: plasma membrane localization protein AtAKT1. Bars = 20 μm.

Subcellular localization of MtCDPK4, 7, 9, 14, 15, 16, 22, and MtCRK6 was analyzed to further explore the role of N-terminal acylation in protein localization. The selected MtCDPKs and MtCRKs covered all groups and types of modifications for N-myristoylation and N-palmitoylation. The results showed that the fluorescence of MtCDPK4, 14, 16, 22, and MtCRK6 was co-localized with plasma membrane localization protein AtAKT1 after plasmolysis, indicating that they are localized on the plasma membrane. MtCDPK7, 9, and 15, which lack one or both N-acylation modification sites, were homogenously disseminated in cytosol and nucleus and highly overlapped with 35S::mCherry and as seen in the result of 35S::GFP signals (Figure 7).

## 3. Discussion

*CDPK* and *CRK* genes are present in photosynthetic organisms and play an important role in plant growth and development [6,8]. Comprehensive genome-wide identification of *CDPK* and *CRK* genes has been widely carried out in some plants, such as 34 *CDPKs* and eight *CRKs* in *Arabidopsis* [9], 31 *CDPKs* and five *CRKs* in rice [41], 29 *CDPKs* and six *CRKs* in tomato [34], 30 *CDPKs* and nine *CRKs* in poplar [42], and 49 *CDPKs* and 14 *CRKs* in *Brassica rapa* [43]. In the present study, six *MtCRK* genes were computationally identified, they and previously identified *MtCDPKs* were named as *MtCDPK1* to *MtCDPK24* and *MtCRK1* to *MtCRK6* based on their chromosomal locations. Compared to previously identified 25 *MtCDPKs*, Doesn’t Make Infections 3 (DMI3, Medtr8g043970) is a well-known protein kinase in *Medicago truncatula*, involving in nodule organogenesis [44]. It belongs to CCaMK, which binds not only calcium but also calmodulin, thus it was not included as CDPK in this study. *MtCDPKs* and *MtCRKs* are not distributed on every chromosome, which is consistent with that in poplar [42], soybean [45], and melon [46].

MtCDPKs and MtCRKs were divided into five groups (CDPK I to IV and CRK) based on the phylogenic tree among those in *Arabidopsis*, rice, and *M. truncatula*, which is consistent with that in other plant species [9,34,41,42,43]. The result indicates that CDPKs and CRKs are ubiquitous and conserved among plant species. MtCDPK and MtCRK members within the same group shared a similar exon-intron structure, intron phases, biochemical properties, and conserved motif compositions, indicating that they are closely evolutionary conservation. Compared to other groups, *MtCRKs* has a similar gene structure with the members in MtCDPK IV, and cluster with the MtCDPK IV group with 100% bootstrap support. Furthermore, the theoretical pI of the members in the MtCRK and MtCDPK IV group tends to be moderately alkaline, while that of in other group shows slightly acidic. The above data are consistent with those in *Arabidopsis*, *Brassica rapa*, and watermelon, indicating that *CRKs* may have evolved from MtCDPK IV [9,43,47]. The members in the same group shared highly similar gene structures and conserved motifs implied that they might have a similar function.

Gene duplication is a primary driving force for plant evolution and leads to the expansion of gene family. Gene duplication models include segmental/whole-genome duplication (WGD), tandem duplication, proximal duplication, transposed duplication, and dispersed duplication [48]. In our study, six pairs of segmental duplications were found in *M. truncatula* and 29 pairs of duplication events were found between *M. truncatula* and *A. thaliana*. Segmental duplication gene pairs were found in *CDPK* and *CRK* gene families in rice [41] and poplar [42]. Inter-genomic duplication events between *Arabidopsis* and other species have been reported. There are 22 collinear blocks of *CDPK* and *CRK* genes between melon and *Arabidopsis* [46]. The variation in the number of *CDPK* among species is associated with species-specific WGD events [6,8]. *M. truncatula* has experienced a high frequency of local genome rearrangement [49]. Our analysis indicated that segmental duplication plays a requisite role in *MtCDPKs* and *MtCRKs* expansion. *Ka/Ks* ratio was used to determine selection pressure on the duplicated gene pairs. The *Ka/Ks* ratio of six paralogous genes was below 0.310. This is consistent with that in tomato [50] and *Brassica rapa* [43]. The result indicated that these pairs have been under strong purifying selection, which may cause limited functional divergence.

Multiple *cis*-acting elements responsible for phytohormones, stresses, growth and development were observed in the promoter regions of *MtCDPKs* and *MtCRKs*, indicating the potential role of *MtCDPKs* and *MtCRKs* in regulating multiple responses to phytohormones, environmental stresses, and development. The spatial expression of *MtCDPKs* and *MtCRKs* displays diversity, for instance, *MtCDPK17* was exclusively expressed in flower, and its homologous genes *AtCPK17* and *AtCPK34* from *Arabidopsis* were reported to be involved in pollen tube growth [51]. *CDPK* and *CRK* family genes are involved in responding to various stresses, such as drought [22,23], salt [26], cold stress [27], and fungus and bacterial pathogens [31]. *MtCDPK22* transcript is specifically and strongly expressed under cold stress, and its homologous gene *OsCPK17* is involved in cold stress response [27]. *MtCDPK8* was highly induced under cold treatment, while its paralogous gene *MtCDPK18* was downregulated expression. Transcripts of *MtCDPK4*, *8*, *16*, *18*, *21*, and *22* were responsive to yeast elicitor treatment. *AtCPK28*, orthologous to *MtCDPK16*, was verified to be a negative regulator of PAMP-triggered immunity (PTI) [52]. The results indicated that MtCDPKs and MtCRKs had functional diversity in *M. truncatula*. *MtCDPK2*, *18*, *19*, and *CRK1*, which contains dehydration-responsive elements (DRE1 or DRE-core), were upregulated under drought stress. In *Arabidopsis*, some AtCPKs are involved in regulating phytohormone and abiotic stresses signaling when specific *cis*-acting elements were detected in the promoter regions [23,53,54]. Therefore, similarities and differences analysis of family members based on the expression pattern and promoter can provide candidate genes for further functional analysis at least in part.

Myristoylation and palmitoylation are unique among lipid modifications [55]. They anchor proteins to membranes through mutual coordination and promote the proteins to exercise physiological processes [56,57,58,59]. An N-myristoylation site and a palmitoylation site are contained in most CDPKs and determine the subcellular distribution of CDPK [58,59]. MtCDPK4, 14, 16, 22, and MtCRK6 harbor both N-myristoylation site and palmitoylation site and were anchored on plasma membrane, while MtCDPK7, 9, and 15 contain no or only one N-acylation site and were distributed in cytosol and nucleus. The results indicated that the N-terminal acylation sites play a key role in the subcellular localization of MtCDPKs and MtCRKs. The subcellular localization of AtCDPK6 and NtCPK5 are also affected by myristoylation/palmitoylation sites mutations [12,60]. Myristoylation-regulated membrane association is loose, while palmitoylation confers stable membrane anchoring. Previous studies have shown that palmitoylation generally occurs at a Cys residue proximal to an N-myristoylation site, indicating that palmitoylation-regulated membrane association might require the participation of N-myristoylation [61,62,63]. In addition, palmitoylation is reversible, which allows membrane-localized proteins to detach from the membrane and translocate to the cytosol or nucleus via thioesterase-mediated de-*S*-palmitoylation [64,65]. Previous reports indicated that subcellular locations of AtCPK10, AtCPK30, and AtCPK32 were changed from plasma membrane to nucleus in response to nitrate [66]. Changes in subcellular localization provide the possibility for CDPKs and CRKs to perform different physiological functions. This study provides a foundation to further explore the functions of MtCDPKs and MtCRKs in *M. truncatula*.

## 4. Materials and Methods

### 4.1. Identification of MtCRK Genes

The sequences of *CRK* genes were obtained from genome databases of *Arabidopsis* (TAIR, http://www.arabidopsis.org/), rice (TIGR, http://rice.tigr.org), and *Medicago truncatula* (http://www.medicagohapmap.org/) respectively. The amino acid sequences of CRKs of *Arabidopsis* and rice were used as reference sequences to search predicted homologs in *Medicago truncatula* using HMMER3.0 Software (http://hmmer.org/). The candidate *CRK* genes were preliminarily identified by screen the gene using threshold of E-value (full sequence and best 1 domain) less than 1E-100. The composition of identified candidate proteins was further verified in SMART databases (http://smart.embl-heidelberg.de/) [67] and NCBI-Conserved Domain database (CDD, https://www.ncbi.nlm.nih.gov/Structure/cdd/wrpsb.cgi), while the sequences with errors, shorter length (<100 aa), and containing incomplete Ser/Thr kinase domain were eliminated. The longest one was chosen for further analysis if a gene had alternative splice variants.

### 4.2. Sequence Analysis of MtCDPKs and MtCRKs

Molecular weight (MW), isoelectronic points (pI), and the grand average of hydropathy (GRAVY) of MtCDPKs and MtCRKs were predicted using The ExPASY PROTPARAM tool (http://web.expasy.org/protparam/). The EF-hands motif was predicted using the ScanProsite tool (https://prosite.expasy.org/prosite.html). The N-terminal myristoylation and palmitoylation sites were predicted using GPS-Lipid 1.0 program with default settings and high threshold [68].

### 4.3. Multiple Sequence Alignments and Phylogenetic Analysis

Multiple alignments of CDPK and CRK protein sequences from *M. truncatula*, *Arabidopsis*, and rice were performed using the MUSCLE program, which has a higher efficiency than CLUSTAL, with default parameters implemented in MEGA7.0 software (https://www.megasoftware.net/). The phylogenetic tree was constructed by using MEGA7.0 with the neighbor-joining method, and the 1000 bootstrap replicated by the Jones, Taylor, and Thornton amino acid substitution model (JTT model) and keeping the other parameters as a default to determine the reliability of the resulting tree [69].

### 4.4. Gene Structure and Conserved Motifs Analysis

The gene structure and conserved motifs were displayed using TBtools software [70]. The conserved motifs of each protein were analyzed using the MEME program (Version 5.1.1) (http://meme-suite.org/tools/meme) [71]. The maximum motif number was set as 20, and the other parameters as default.

### 4.5. Chromosomal Location and Synteny Correlation Analysis

For analysis of chromosomal locations of *MtCDPK* and *MtCRK* genes, the Circos diagram was illustrated by annotating genes on their specific chromosomal position in the genome annotation using TBtools software. The syntenic gene relationships between the homologs of *A. thaliana* and *M. truncatula* were verified and visualized using the Circos tool implemented in TBtools software.

### 4.6. Calculation of Ka/Ks Ratios of Paralogous Gene Pairs of MtCDPKs and MtCRKs

The nonsynonymous (*Ka*) and synonymous (*Ks*) substitution rates of paralogous gene pairs of *MtCDPKs* and *MtCRKs* were calculated based on the standard genetic codon table using the Nei–Gojobori method (Jukes–Cantor model) in MEGA 7.0 [69].

### 4.7. Analysis of Cis-Acting Regulatory Elements in Promoter Regions

A 2.0 kb of promoter sequence upstream from the transcription start site in each *MtCDPK* and *MtCRK* was extracted from the *M. truncatula* genome database and analyzed using PlantCARE online software (http://bioinformatics.psb.ugent.be/webtools/plantcare/html/) to predict the putative cis-acting regulatory elements [72].

### 4.8. Analysis of Expression Profiles of MtCDPKs and MtCRKs

Microarray data on the expression profile of *MtCDPKs* and *MtCRKs* in roots, stem, vegetative bud, leaf, petiole, flower, pods, and seeds and responses to pathogen were extracted from the MtGEA (https://mtgea.noble.org/v3/) [73]. The probe with the maximum value was selected for the subsequent analysis when a gene corresponds to multiple probes. The microarray data were normalized based on the mean expression value of each gene in all organs analyzed, while the normalized data were used to generate the heatmap using the TBtools software. The expression pattern of *MtCDPKs* and *MtCRKs* in response to cold, drought, and salt stresses was from NCBI GEO with the dataset accession of GSE136739 [74]. FPKM was used for representing the expression abundance of each *MtCDPK* and *MtCRK* gene. The relative transcript level after treatments was calculated compared with the control before treatment (0 h). The clustered heatmap was portrayed based on the relative expression using the TBtools software.

### 4.9. Analysis of Subcellular Localization

The coding sequence without the terminal codon of selected MtCDPKs and MtCRKs was amplified using specific primer pairs and inserted into the expression vector pCAMBIA1305-GFP driven by CaMV 35S promoter utilizing homologous recombination (Table S8). *Agrobacterium tumefaciens* EHA105 harboring the constructed vectors or empty vector in combination with 35S::mCherry vector or 35S::AtAKT1-mCherry vector were used to co-transform the abaxial surfaces of leaves of 4-week-old *N. benthamiana*. Then the tobaccos were grown under normal condition. Before the observation, the tobacco injected with P.M-marker was immersed in 0.3 g/mL sucrose solution for plasmolysis. Fluorescence was observed using confocal laser scanning microscopy (Zeiss LSM800, Germany) at 72 h after transformation.

## Figures and Tables

**Table 1:** Characteristics of *CDPK* and *CRK* genes in *Medicago truncatula.*

Gene Name	Gene ID	Chromosome Location	No. of aa	MW (kDa)	pI	GRAVY	No. of EF Hands	*N*-Acylation Prediction (No.)
*MtCDPK1*	Medtr0028s0170.1	scaffold0028:72630..76559	534	59.85	5.65	−0.542	4	*N*-Myr (1)–Palm (2)
*MtCDPK2*	Medtr1g026190.1	chr1:8404402..8408507	533	60.47	6.21	−0.591	4	*N*-Myr (1)–Palm (1)
*MtCDPK3*	Medtr1g041150.1	chr1:18398807..18403890	581	65.08	5.37	−0.376	4	*N*-Myr (1)–Palm (1)
*MtCDPK4*	Medtr1g052530.1	chr1:21386135..21390691	528	60.13	6.16	−0.501	4	*N*-Myr (1)–Palm (2)
*MtCDPK5*	Medtr1g054865.1	chr1:24041840..24046332	607	66.45	5.51	−0.334	4	*N*-Myr (1)–Palm (1)
*MtCDPK6*	Medtr1g055255.1	chr1:24417898..24422244	589	66.07	6.52	−0.478	4	*N*-Myr (2)–Palm (1)
*MtCDPK7*	Medtr1g096490.1	chr1:43462266..43465665	499	56.18	5.21	−0.380	4	*N*-Palm (1)
*MtCDPK8*	Medtr3g051770.1	chr3:20500557..20506458	538	60.60	5.77	−0.460	4	*N*-Myr (1)–Palm (1)
*MtCDPK9*	Medtr3g098070.1	chr3:44756354..44761409	495	55.92	5.17	−0.342	4	- (0)
*MtCDPK10*	Medtr3g098090.1	chr3:44766535..44770211	498	56.37	5.36	−0.395	4	- (0)
*MtCDPK11*	Medtr4g066660.1	chr4:25214155..25217596	545	62.02	6.06	−0.549	4	*N*-Myr (1)–Palm (1)
*MtCDPK12*	Medtr4g107490.1	chr4:44509014..44514153	555	63.02	6.37	−0.470	4	*N*-Myr (1)–Palm (2)
*MtCDPK13*	Medtr4g132040.1	chr4:55151186..55153959	543	60.88	5.83	−0.415	4	*N*-Myr (1)–Palm (1)
*MtCDPK14*	Medtr4g132070.1	chr4:55167475..55172407	539	60.56	6.16	−0.463	4	*N*-Myr (1)–Palm (1)
*MtCDPK15*	Medtr5g009830.1	chr5:2478209..2483414	517	58.73	6.44	−0.534	4	*N*-Myr (1)
*MtCDPK16*	Medtr5g022030.1	chr5:8628890..8634949	560	63.50	9.10	−0.615	4	*N*-Myr (1)–Palm (1)
*MtCDPK17*	Medtr5g089320.1	chr5:38807982..38811203	523	58.50	5.43	−0.535	4	*N*-Myr (1)–Palm (2)
*MtCDPK18*	Medtr5g092810.1	chr5:40530948..40535829	540	61.32	7.67	−0.615	4	*N*-Myr (2)–Palm (1)
*MtCDPK19*	Medtr5g099240.1	chr5:43501966..43509933	559	62.93	5.68	−0.443	4	*N*-Myr (1)–Palm (1)
*MtCDPK20*	Medtr7g068710.1	chr7:25219104..25225477	530	59.97	6.03	−0.457	4	*N*-Myr (1)–Palm (2)
*MtCDPK21*	Medtr7g091890.1	chr7:36380965..36385094	533	60.59	6.16	−0.554	4	*N*-Myr (1)–Palm (2)
*MtCDPK22*	Medtr7g106710.1	chr7:43452426..43456560	602	68.15	5.34	−0.451	4	*N*-Myr (1)–Palm (1)
*MtCDPK23*	Medtr8g095440.1	chr8:39939663..39944572	503	56.82	5.20	−0.344	4	- (0)
*MtCDPK24*	Medtr8g099095.1	chr8:41618323..41623707	516	58.06	5.93	−0.410	4	*N*-Myr (1)–Palm (1)
*MtCRK1*	Medtr3g092230.1	chr3:42122948..42129657	588	65.61	8.74	−0.318	0	*N*-Myr (1)–Palm (2)
*MtCRK2*	Medtr4g086660.1	chr4:33966651..33972436	592	66.57	9.07	−0.398	0	*N*-Myr (2)–Palm (1)
*MtCRK3*	Medtr5g017830.1	chr5:6589564..6594550	587	65.86	8.49	−0.309	2	*N*-Myr (1)–Palm (1)
*MtCRK4*	Medtr7g089480.1	chr7:35036401..35042624	595	66.80	8.84	−0.305	0	*N*-Myr (1)–Palm (1)
*MtCRK5*	Medtr7g118020.1	chr7:48973096..48979529	596	66.62	8.92	−0.415	3	*N*-Myr (1)–Palm (1)
*MtCRK6*	Medtr8g079790.1	chr8:34200112..34204254	581	65.15	9.00	−0.309	1	*N*-Myr (1)–Palm (1)

**Table 2:** Duplications of the *CDPK* and *CRK* genes in *Medicago truncatula.*

Gene 1	Gene 2	*Ka*	*Ks*	*Ka/Ks*	Purifying Selection	Duplicate Type
*MtCDPK6*	*MtCDPK22*	0.243	0.791	0.307	Yes	Segmental
*MtCDPK8*	*MtCDPK18*	0.112	0.659	0.171	Yes	Segmental
*MtCDPK9*	*MtCDPK23*	0.100	0.720	0.139	Yes	Segmental
*MtCDPK15*	*MtCDPK24*	0.098	0.768	0.128	Yes	Segmental
*MtCRK2*	*MtCRK3*	0.168	1.936	0.087	Yes	Segmental
*MtCRK3*	*MtCRK6*	0.136	0.708	0.192	Yes	Segmental

## Data Availability

The data that support the findings of this study are available from the corresponding author upon reasonable request.

## Supplementary Materials

The following are available online at https://www.mdpi.com/1422-0067/22/3/1044/s1.
