# Supplementary material for: Genome-Wide Identification and Characterization of Calcium-Dependent Protein Kinase (CDPK) and CDPK-Related Kinase (CRK) Gene Families in Medicago truncatula
Source: Int J Mol Sci. 2021 Jan 21;22(3):1044. doi: 10.3390/ijms22031044 (PMC7864493; doi:10.3390/ijms22031044)
Supplement: Supplementary file 1 [file ijms-22-01044-s001.zip › Figure S1.docx]

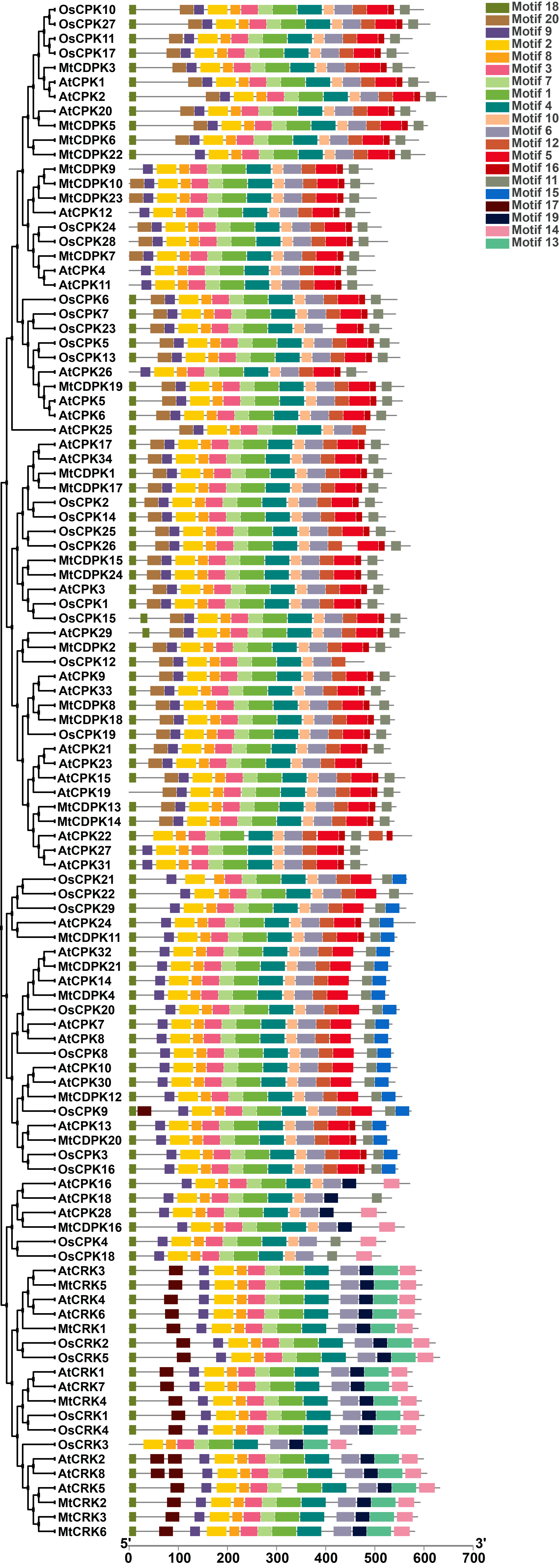


Figure S1. The conserved motifs of CDPK and CRK proteins from *M. truncatula*, *Arabidopsis*, and rice were identified using MEME. The motifs were indicated by different colored boxes and their numbers were listed on the right.
